# Phylogeography of *Tibouchina papyrus* (Pohl) Toledo (Melastomataceae), an endangered tree species from rocky savannas, suggests bidirectional expansion due to climate cooling in the Pleistocene

**DOI:** 10.1002/ece3.236

**Published:** 2012-05

**Authors:** Rosane Garcia Collevatti, Thaís Guimarães de Castro, Jacqueline de Souza Lima, Mariana Pires de Campos Telles

**Affiliations:** Laboratório de Genética e Biodiversidade, Departamento de Biologia Geral, ICB, Universidade Federal de GoiásCxP. 131, 74001-970 Goiânia, GO, Brazil

**Keywords:** Cerrado rupestre, climatic relict, coalescent analysis, cpDNA, disjunct geographical distribution, microsatellites

## Abstract

Many endemic species present disjunct geographical distribution; therefore, they are suitable models to test hypotheses about the ecological and evolutionary mechanisms involved in the origin of disjunct distributions in these habitats. We studied the genetic structure and phylogeography of *Tibouchina papyrus* (Melastomataceae), endemic to rocky savannas in Central Brazil, to test hypothesis of vicariance and dispersal in the origin of the disjunct geographical distribution. We sampled 474 individuals from the three localities where the species is reported: Serra dos Pirineus, Serra Dourada, and Serra de Natividade. Analyses were based on the polymorphisms at cpDNA and on nuclear microsatellite loci. To test for vicariance and dispersal we constructed a median-joining network and performed an analysis of molecular variance (AMOVA). We also tested population bottleneck and estimated demographic parameters and time to most recent common ancestor (TMRCA) using coalescent analyses. A remarkable differentiation among populations was found. No significant effect of population expansion was detected and coalescent analyses showed a negligible gene flow among populations and an ancient coalescence time for chloroplast genome. Our results support that the disjunct distribution of *T. papyrus* may represent a climatic relict. With an estimated TMRCA dated from ∼836.491 ± 107.515 kyr BP (before present), we hypothesized that the disjunct distribution may be the outcome of bidirectional expansion of the geographical distribution favored by the drier and colder conditions that prevailed in much of Brazil during the Pre-Illinoian glaciation, followed by the retraction as the climate became warmer and moister.

## Introduction

The understanding of the ecological and evolutionary mechanisms involved in the origin of disjunct distributions in plant species has been improved by phylogeographic analyses (e.g., Azuma et al. 2001; Karanth 2003; Gaudeul 2006; Collevatti et al. 2009a). While for some species the disjunct distribution represents a climatic relict of the ice ages, caused by the range contraction in an ancient more widely distribution (e.g., Stehlik et al. 2000; Collevatti et al. 2009a; Lorenz-Lemke et al. 2010), long distance dispersal to new suitable habitats may also be responsible for disjunct distributions (e.g., Dick et al. 2003, 2007; Gaudeul 2006). Also, a number of studies based on the fossil pollen record now available for the Neotropics show that the distribution and composition of vegetation were deeply influenced by the climatic oscillations of the Tertiary and Pleistocene.

During the early Holocene period [until ca. 6000–5000^14^C year BP (before present)], the climate was drier in most of the South American savannas and distribution of savanna-like vegetation in Central and Southeast Brazil was more extensive in early compared with the late Holocene (Salgado-Laboriau et al. 1998; Behling and Hooghiemstra 2000; Behling 2003). In southeastern Brazil, drier climates lasted until 8000–8500^14^C year BP or later (Behling 1995, 2003) and savanna landscapes were dominated by grasslands and frequent fires were recorded. The current cerrado vegetation exist in the region only in the latest Holocene period (since 970 or 600 year BP for some regions) under the current wet climatic conditions, with an annual dry season of about 4 months. The arid period seems to be a widespread event in South America, although the precise age may differ due to latitude (Ledru 1993; Salgado-Laboriau et al. 1998; Behling 2003). Hence, the fossil record shows that savanna expansion during glacial periods of the Quaternary was characterized mainly by an open savanna with species more adapted to drier and highly seasonal climate. In such a dynamic scenario, demographic expansion and retraction of populations will lead to loss in genetic variation and increase in homozygosity, because only some genotypes may survive and expand in each new cycle (Hewitt 1996; Davis and Shaw 2001). Also, signal of bottleneck events may be found in extant populations due to retraction during interglacial periods, such as unimodal mismatching distribution (Excoffier 2004). Because expectation to time to most recent common ancestor (TMRCA) is directly related to number of genes in population and probability of coalescence is inversely related to the number of genes in population (Kingman 1982a, b), most coalescences will occur before demographic expansion, when population size was small (see Excoffier et al. 2009 for a review). Thus, for savanna species adapt to drier and colder climates of glacial periods, it is expected that TMRCA coincides with interglacial periods when populations became restricted.

Although a number of studies on phylogeography of Neotropical plant species are now available to clarify how climatic oscillations in Tertiary/Quaternary may have affected species distribution in the Brazilian savannas, these studies focus mainly on widely distributed species (e.g., Collevatti et al. 2003; Ramos et al. 2007). Only one study was performed with an endemic species restricted to rocky savannas from the Brazilian Cerrado Biome (Collevatti et al. 2009a). Many endemic species from rocky savannas present disjunct geographical distribution and like so are suitable models to test hypotheses about the ecological and evolutionary mechanisms involved in the origin of disjunct distributions in these habitats.

Rocky savannas (cerrado rupestre) are particular vegetation communities associated to outcrops of sandstone and quartzite soils of the Brazilian Shield in the Cerrado Biome in Central and Southeast Brazil, with high number of endemic species and high influence of fire during the dry season (Ribeiro and Walter 1998). It is found mainly in Serra do Espinhaço, Southeast and Northeast Brazil, and in some highlands in Central Brazil such as on Serra dos Pirineus, Chapada dos Veadeiros, Serra Dourada, and Serra dos Cristais in Goiás (GO), in Serra Geral in Tocantins (TO), Chapada dos Guimarães in Mato Grosso, MT, and in similar scattered habitats in Chapada da Pratinha (GO and Federal District (for detailed description, see Furley and Ratter 1988).

**Figure 1:**
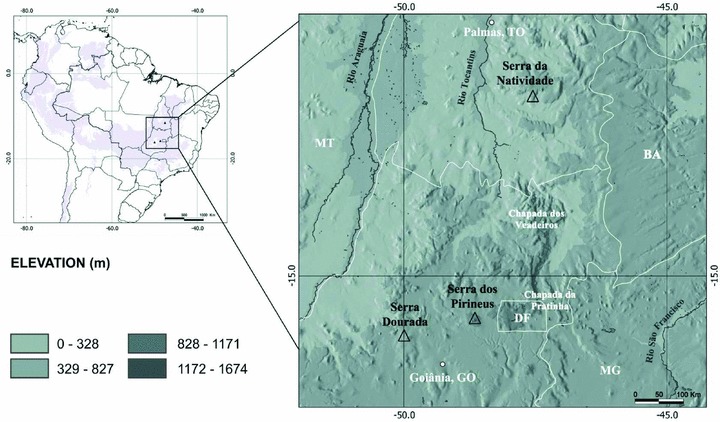
The sample sites of *Tibouchina papyrus* populations in Central Brazil. NAT, Serra da Natividade, Natividade, TO; PIR, Serra dos Pirineus (PIR), Pirenópolis, GO; SDO, Serra Dourada, Cidade de Goiás, GO. White lines are the state divisions. Black lines are the main rivers in the studied region.

*Tibouchina papyrus* (POHL) Toledo (Melastomataceae) is an endemic tree species from outcrop quartzite and sandstone of the rocky savannas (cerrado rupestre) of the Cerrado biome. Flowers are purple and buzz-pollination is performed mainly by large bees, such as *Xylocopa* spp., *Bombus* spp., and *Centris* spp. (Montoro and Santos 2007) and the very small seeds are dispersed by autochory. It is considered a vulnerable species in the IUCN Red List (IUCN 2001) and also by the Brazilian Environment Ministry (see http://www.biodiversitas.org.br/florabr/lista_florabr.pdf) due to its natural endemism and habitat vulnerability. The species is reported only in three localities in the Brazilian Shield, Central Brazil (Fig. 1) in Serra dos Pirineus and Serra Dourada, Goiás (GO), and in Serra da Natividade, Tocantins (TO) (Teixeira 1969; Montoro and Santos 2007).

In the present work, we aimed to understand the origin of the disjunct geographical distribution of *T. papyrus.* For this, we used a phylogeographical approach to test the hypothesis that the present disjunct geographical distribution is a climatic relict of the ice ages of the Tertiary/Quaternary, due to range contraction of a previously more widely distributed species caused by the changes in climatic conditions that affected suitable habitat distribution. If our hypothesis holds, we predicted a high differentiation among populations at both chloroplast genome and microsatellite loci, no recent gene flow among populations, an ancient TMRCA and a signal of bottleneck during interglacial periods. The analyses were based on the polymorphism at three noncoding chloroplast DNA regions and on 10 nuclear microsatellite loci. For plants, the comparative analysis of nuclear and organelle genomes, with different modes of inheritance and mutation and evolutionary rates, may clarify the relative importance of pollen and seed flow on population structure and provide additional insights about the evolution and historical spread of populations (McCauley 1995; Schaal et al. 1998; Collevatti et al. 2003, 2009a, b). Additionally, because of the haploid nature and mode of inheritance, the effective population size of the chloroplast genome is one-half the size of the nuclear genome, leading to a stronger effect of genetic drift on population genetic structure (Ennos 1994).

## Material and Methods

### Populations, sampling, and DNA extraction

We sampled *T. papyrus* populations in each of the three localities where the species is reported: Serra dos Pirineus (PIR) and Serra Dourada (SDO), Goiás (GO), and in Serra da Natividade (NAT), Tocantins (TO) (Fig. 1). Notice that these small number of known local populations is not due to lack of knowledge (i.e., the Wallacean shortfall), because extensive sampling of Cerrado plants and rocky savannas were performed in the last years, searching in many cases especially for this species. Because *T. papyrus* is locally distributed in well-delimited patches, we sampled all individuals visualized in PIR (216) and SDO (66). In population NAT, we randomly sampled 192 individuals because this population was the largest, with ∼1500 individual trees. For each individual, expanded leaves were sampled and stored at –80°C. DNA extraction followed the standard CTAB procedure (Doyle and Doyle 1987). Distances between pairs of populations were (Fig. 1): NAT–PIR, 554.82 km; NAT–SDO, 472.32 km; PIR–SDO, 146.97 km.

### Sequencing analyses

For sequencing analyses, we randomly chose 32 adult individuals in each population. Three noncoding regions of the chloroplast genome (cpDNA) were sequenced: the region between the genes *psbA* and *trnH* (Azuma et al. 2001), *trnS* and *trnG* (Shaw et al. 2005), and *trnC* and *ycf6* (Demesure et al. 1996). Fragments were amplified by polymerase chain reaction (PCR) in a 20 μL volume containing 1.0 µM of each primer, one unit Taq DNA polymerase (Phoneutria, Belo Horizonte, BR), 250 µM of each dNTP, 1× reaction buffer (10 mM Tris–HCl, pH 8.3, 50 mM KCl, 1.5 mM MgCl_2_), 250 µg of BSA, and 6.0 ng of template DNA. Amplifications were performed using a GeneAmp PCR System 9700 (Applied Biosystems, Foster City, California, USA) with the following conditions: 96°C for 2 min (1 cycle); 94°C for 1 min, annealing temperature for 1 min (55^o^C for *psbA-trnH*, 56^o^C for *trnS–trnG*, 60^o^C for *trnC-ycf6*), 72°C for 1 min (30 cycles); and 72°C for 10 min (1 cycle). Polymerase chain reaction products were sequenced on an ABI 3100 automated DNA sequencer (Applied Biosystems, CA) using the DYEnamic™ ET terminator cycle sequencing kit (GE HealthCare, Uppsala, Sweden), according to manufacturer's instructions. All fragments were sequenced in forward and reverse directions.

Sequences were aligned using CLUSTALX (Thompson et al. 1997), and characters (each base pair) were equally weighted before analysis. For statistical analyses, the data of all sequenced regions were concatenated.

Populations were characterized for nucleotide (π) and haplotype (*h*) diversity (Nei 1987). Parameters were estimated using the software Arlequin v. 3.11 (Excoffier et al. 2005). To understand the origin of disjunct distribution and test the hypothesis of vicariance, we performed the following statistical analyses.

First, we generated a hypothesis of phylogenetic relationship among haplotypes using the median-joining network analysis based on parsimony criteria (Bandelt et al. 1999), implemented in the software Network 4.2.0.1 (Forster et al. 2004). Then, an analysis of molecular variance (AMOVA, Excoffier et al. 1992) was performed using Arlequin, to test the hypothesis of genetic differentiation among populations. The parameter *F*_ST_ and pairwise *F*_ST_ between all pairs of subpopulation was estimated from AMOVA. Significance of *F*_ST_ and pairwise *F*_ST_ were tested by a nonparametric permutation test (Excoffier et al. 1992) implemented in the Arlequin software. Then, the hypothesis that the current pattern of haplotype diversity and distribution was caused by contraction of an ancient widely distributed population was tested under the assumption of a bottleneck followed by a sudden expansion. Bottleneck analyses were performed under two assumptions: (1) overall populations, under the assumption that *T. papyrus* was more widely distributed in Central Brazil during glaciations, and that the present disjunct distribution is derived from the contraction of an ancient population (climatic relict); (2) for each population, based on network relationships and high differentiation among populations (see results below) under the assumption that, after the species has attained the disjunction distribution, populations have undergone independent demographic histories, without gene flow among them. The mismatch distribution was obtained and the hypothesis of expansion was tested using the Harpending´s Raggeness Index R (Roger and Harpending 1992; Schneider and Excoffier 1999) and Tajima's D test of neutrality (Tajima 1989) also using Arlequin software.

Finally, a coalescent model (Kingman 1982a, b) was used to estimate demographic parameters and TMRCA. The demographic parameters θ= 2*µN_e_* (coalescence force or mutation parameter), *g* (growth force or exponential growth rate), where *θ_t_*=θ_now_ exp(–*gtµ*) and *t* is time in mutational unit, and *M*= 2*N_e_m*/θ (migration force or immigration rate) were estimated based on Bayesian estimation using Markov Chain Monte Carlo approach implemented in LAMARC 2.1.5 software (Kuhner et al. 2006). Four independent analyses were run with 10 initial chains and two final chains and combined results were generated using Tracer v. 1.5 (Rambaut and Drummond 2007). Most probable estimates (MPE) were obtained, that is, the highest point on the posterior probability curve for a given parameter, which is the best solution found by a Bayesian run, and also the confidence interval around the estimate of each parameter (Kuhner and Smith 2007). Time to most recent common ancestor (TMRCA) was estimated based on Bayesian phylogenetic analysis implemented in BEAST 1.6.1 software (Drummond and Rambaut 2007). A relaxed molecular clock was assumed (uncorrelated lognormal), and constant population size (based on the results on population expansion, see below). Four independent analyses were run for 10^8^ generations under HKY+G substitution model. Convergence and stationarity were checked using the software Tracer v1.5 (Rambaut and Drummond 2007). To estimate TMRCA in years, we used mutation rates previously estimated for chloroplast noncoding regions (Yamane et al. 2006). Yamane and coworkers estimated an indel mutation rate of 0.8 ×10^–9^± 0.04 × 10^–9^ per nucleotide per year and 1.52 × 10^–9^± 0.06 × 10^–9^ for substitution mutation rate. As the chloroplast regions used in the present work may have both indels and substitutions we estimated TMRCA using each rate and then combined results using Tracer v. 1.5. The substitution rates selected here are conservative, lower than the fastest observed rates for the chloroplast genome (e.g., Wolfe et al. 1987). Higher rates would imply younger dates for the splitting of the lineages but also the published cpDNA mutation rates are based on grass and herbs, which may present twofold faster rates of substitution than woody plants (e.g., Clegg et al. 1994; Smith and Donoghue 2008) leading to an overestimation of divergence times. However, we acknowledge a potential limitation of the method, which may lead to overestimation of the splitting dates because the most recent common ancestor (MRCA) of the haplotypes (their coalescent) does not necessarily correspond to the real temporal split of the populations but may precede the actual divergence of the populations (Arbogast et al. 2002).

### Microsatellite analyses

For microsatellite analysis, all individuals in each locality (474) were genotyped for 10 microsatellite loci, previously developed for *Tibouchina papyrus* (Telles et al. 2011). Microsatellite amplifications were performed in a 15 μL volume containing 3.96 µM of each primer, one unit Taq DNA polymerase (Phoneutria, BR), 250 µM of each dNTP, 1× reaction buffer (10 mM Tris–HCl, pH 8.3, 50 mM KCl, 1.5 mM MgCl_2_), and ∼12.5 ng of template DNA. Amplifications were performed using a PE 9700 Thermal Controller (Applied Biosystems, CA) with the following conditions: 94°C for 5 min (1 cycle), 94°C for 1 min, 54°C–66°C for 1 min (according to each locus, see Telles et al. 2011), 72°C for 1 min (30 cycles); and 72°C for 7 min (1 cycle). The amplified products were separated on 6% denaturing polyacrylamide gels stained with silver nitrate (Creste et al. 2001) and sized by comparison to a 10-bp DNA ladder standard (Invitrogen). All individuals were genotyped at least two times in independent PCR amplifications and polyacrylamide gels to avoid genotyping error. Although no information on chromosome number or ploidy level are currently available for *T. papyrus*, and a high variation has been described for the genus (e.g., 2*n*= 18 ∼ 2*n*= 56, see Molero et al. 2006), microsatellite loci segregation followed the pattern expected for diploid species. Thus, the analyses were not affected by the ploidy level.

Populations were characterized for number of alleles per locus, allelic richness based on rarefaction method (see Hurlbert 1971; Mousadik and Petit 1996; Petit et al. 1998), and observed and expected heterozygosities under Hardy–Weinberg equilibrium (Nei 1978), and inbreeding coefficients (*f*). Analyses were performed with FSTAT 2.9.3.2 (Goudet 2002) and randomization based tests with Bonferroni correction were performed for deviation from Hardy–Weinberg expectations and linkage equilibrium (Goudet et al. 1996).

Genetic differentiation among populations was assessed by Wright's *F*-statistics, *F*_IT_, *F*_ST_, and *F*_IS_ (Wright 1951), obtained from an analysis of variance of allele frequencies (Cockerham 1969; Weir and Cockerham 1984), implemented in FSTAT 2.9.3.2 (Goudet 2002). To verify the contribution of stepwise-like mutations to the genetic differentiation, we estimated Slatkin's *R*_ST_ (Slatkin 1995) obtained from an analysis of variance of allele size following Goodman (1997). Then, we tested the hypothesis that *F*_ST_=*R*_ST_ based on Hardy et al. (2003), using the software SPAGeDI (Hardy and Vekemans 2002). The comparison of *F*_ST_ and *R*_ST_ provides insights into the role of drift and mutation in population differentiation, because *R*_ST_ is expected to be larger than *F*_ST_ under stepwise-like mutations, but equal when differentiation is caused only by drift (see Hardy et al. 2003).

The effect of strong reduction in population size due to bottleneck in current population diversity was analyzed using the Wilcoxon sign-rank test (Luikart et al. 1998) implemented in Bottleneck software (Cornuet and Luikart 1997). The bottleneck process may cause a faster loss of heterozygosity under mutation–drift equilibrium (*H*_eq_) than loss of observed heterozygosity (*H*_o_). Hence, populations that have experienced recent reduction in effective population size may present higher *H*_o_ than *H*_eq_ for a given number of alleles in the population (Maruyama and Fuerst 1985). The analyses were run under the assumption of the three models of mutation to verify the sensitivity of the results to the mutation model (see Cornuet and Luikart 1997): IAM (Infinite Allele Model), SMM (Stepwise Mutation Model), and TPM (Two-phase Mutation Model).

Demographic parameters were estimated for microsatellite data based on the coalescent model as described above for sequencing data, correcting for diploid genome (θ= 4*µN_e_* and *M*= 4*N_e_m*/θ), using LAMARC software. Time to most recent common ancestor (TMRCA) was estimated from overall θ. We used the lowest mutation rate reported for microsatellite markers in plants, 2.4 × 10^–4^ mutation per allele per generation (95% CI = [1.4 × 10^–4^; 4.2 × 10^–4^]) (Thuillet et al. 2002), often quoted in the range of 10^–3^ to 10^–4^ per locus per generation (e.g., Ellegren 2000; Udupa and Baum 2001; Thuillet et al. 2002; Vigouroux et al. 2002; Marriage et al. 2009). We chose the lowest value based on the comparison of *F*_ST_ and *R*_ST_ (see results below) and used a generation time of eight years (MPC Telles, unpublished data).

## Results

### Sequencing analyses

Amplification of the noncoding regions *psbA-trnH, trnS-trnG*, and *trnC-ycf6* generated fragments of 268 bp, 580 bp, and 294 bp, respectively. All sequences were submitted to GenBank database (accession numbers: JN969604 to JN969699, for *psbA-trnH*, JN969700 to JN969795, for *trnC-ycf6*; JN969796 to JN969892, for *trnS-trnG*).

For the 96-sequenced individuals, *psbA-trnH* noncoding region presented eight different haplotypes, with five polymorphic sites. For the region *trnC-ycf6*, we found three haplotypes and two polymorphic loci. For *trnS-trnG,* we found five haplotypes and four polymorphic sites (Table S1). Gene diversity for *psbA-trnH* (*h*= 0.771) was very similar to the value found for *trnS-trnG* (*h*= 0.775) and nucleotide diversity (π= 0.0046; SD = 0.0033) was higher than for *trnS-trnG* (π= 0.0025; SD = 0.0017). The region *trnC-ycf6* presented the lowest level of gene diversity (*h*= 0.341) and nucleotide diversity (π= 0.0023; SD = 0.0019). The combined data from the three sequence regions generated a fragment of 1142 bp, with 17 haplotypes, and 11 polymorphic sites (Table S1).

**Figure 2:**
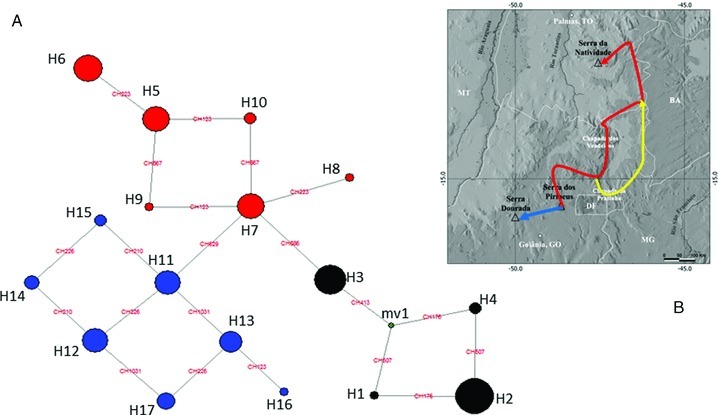
(A) Median-joining network based on the sequences of chloroplast noncoding regions from 96 individuals of *Tibouchina papyrus* from three localities in Central Brazil. Circumference size is proportional to the haplotype frequency. Haplotypes corresponds to those described in S1. All mutations are shown in the network, mv1, median vector. Different colors were assigned for each population: NAT, black; PIR, red; SDO, blue. (B) The model of bidirectional expansion of ancestral population from PIR to SDO and NAT. The arrows show the possible paths following the hills with two possible paths from PIR to NAT.

Population SDO presented the highest genetic diversity (Table 1), followed by PIR. However, nucleotide diversity was very similar among populations (Table 1). Populations did not share any haplotype (Fig. 2). Haplotype H07 from population PIR was the most ancient (Fig. 2), but H02 was the most frequent haplotype.

**Table 1:** Genetic diversity and demographic parameters for *Tibouchina papyrus* populations based on Bayesian coalescence analysis for combined chloroplast DNA data from *psbA-trnH*, *trnS-trnG*, and *trnC-ycf6*. *N*= 32 for all populations (Pop). *k*–number of haplotypes; *h*–haplotype diversity; π–nucleotide diversity; SD–standard deviation; θ–coalescent parameter; SE–standard error; *g*–exponential growth parameter (values followed by ns did not differ from zero based on the confidence interval around the estimate).

	Combined cpDNA				
Pop	*k*	*h*	π (±SD)	θ (SE)	*g* (SE)
NAT	4	0.591	0.0013 ± 0.0009	7.83E–05 (7.39E–06)	309.839^ns^ (46.7671)
PIR	6	0.762	0.0012 ± 0.0009	5.09E–05 (3.24E–06)	322.301^ns^ (100.1479)
SDO	7	0.837	0.0012 ± 0.0008	1.14E–04 (7.54E–06)	353.308^ns^ (113.5003)
Overall	17	0.912	0.0029 ± 0.0017	2.77E–04 (1.16E–06)	394.452^ns^ (57.9844)

Analysis of molecular variance showed a high differentiation among populations (*F*_ST_= 0.684, *P* < 0.001), and pairwise *F*_ST_ were significant and high for all pairs of populations (NAT–PIR, *F*_ST_= 0.821, *P* < 0.001; NAT–SDO, *F*_ST_= 0.802, *P* < 0.001; PIR–SDO, *F*_ST_= 0.755, *P* < 0.001).

Roger–Harpending mismatch distribution test and Tajima's neutrality test did not show any significant effect of population expansion after a bottleneck (*P* > 0.05 for all populations and overall populations). Coalescent analysis performed with LAMARC software also showed that populations are not expanding but have constant size with very low mutation parameter θ (Table 1), indicating a historical low effective population size or a low mutation rate. Number of migrants per generation was negligible for all pairwise comparisons (Table 2).

**Table 2:** Number of migrants per generation for *Tibouchina papyrus* populations, based on Bayesian coalescence analysis for combined cpDNA data from *psbA-trnH*, *trnS-trnG*, and *trnC-ycf6*, and on 10 microsatellite loci. Migration direction is from populations (Pop) in the lines into populations in the columns.

	Combined cpDNA			Microsatellites		
Pop	NAT	PIR	SDO	NAT	PIR	SDO
NAT	–	0.0137	0.0091	–	0.0048	0.0007
PIR	0.0184	–	0.0079	0.0574	–	0.0041
SDO	0.0076	0.0054	–	0.0014	0.0007	–

**Figure 3:**
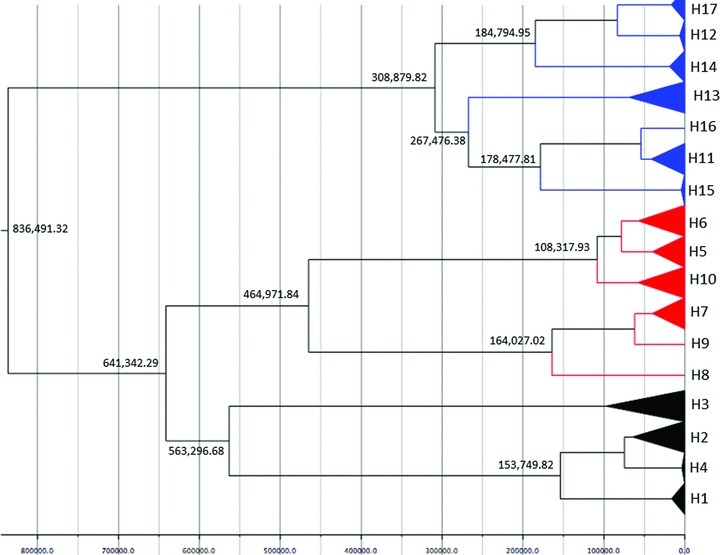
Relationships and TMRCA (time to most recent common ancestor) of the lineages from 96 individuals of three populations of *Tibouchina papyrus* in Central Brazil, based on Bayesian coalescent analyses of chloroplast sequences. Haplotypes corresponds to those described in Table S1. Different colors were assigned for each population: NAT, black; PIR, red; SDO, blue. Time scale is in years before present.

Coalescent analyses performed with BEAST software indicated an ancient TMRCA for *T. papyrus,* dated from 836.491 year BP ± 107.515 year BP for chloroplast genome (Fig. 3). The ancestral diverged in two major lineages that originated population SDO and the ancestral of PIR and NAT populations (Fig. 3). The last lineage, diverged at 661,342.29 ± 100.515 year BP.

### Microsatellite analyses

All pairs of microsatellite loci were in linkage equilibrium (all *P* > 0.05). Genetic diversity was higher at population NAT, but allelic richness was higher in SDO (Table 3). Populations PIR and SDO presented observed heterozygosities statistically lower than expected under Hardy–Weinberg equilibrium (Table 3).

**Table 3:** Genetic diversity and demographic parameters for *Tibouchina papyrus*, based on the polymorphism at 10 microsatellite loci. *N*–number of individuals analyzed; *A_r_*–allelic richness; *H_e_*–expected heterozygosity; *H*_o_–observed heterozygosity; *f*–inbreeding coefficient (values followed by ns are not significant, for *P*= 0.00167, Bonferroni correction for a nominal value of 0.05); θ–coalescent parameter; SE–standard error; *g*–exponential growth parameter (values followed by ns did not differ from zero based on the confidence interval around the estimate).

Population	*N*	*A_r_*	*H_e_*	*H*_o_	*f*	θ (SE)	*g* (SE)
NAT	192	1.8	0.409	0.386	0.057^ns^	0.015 (0.0003)	999.896 (1.1061)
PIR	216	1.5	0.205	0.155	0.246	0.009 (0.0005)	999.981 (0.1034)
SDO	66	2.1	0.357	0.280	0.216	0.019 (0.0009)	–56.648^ns^ (5.4947)
Overall	474	–	–	–	–	1.505 (0.0007)	5.433 (1.3657)

Populations were highly differentiated (*F*_ST_= 0.712, *P*= 0.002). *R*_ST_ also showed a high level of genetic differentiation (*R*_ST_= 0.831, *P* < 0.001), but *F*_ST_ and *R*_ST_ were not significantly different (*P*= 0.427). A significant amount of inbreeding was found (*F*_IS_= 0.127, *P*= 0.002), and nonrandom mating among populations (*F*_IT_= 0.748, *P*= 0.002). Pairwise *F*_ST_ were also significant and high for all pairs of populations (NAT–PIR, *F*_ST_= 0.843, *P* < 0.017; NAT–SDO, *F*_ST_= 0.691, *P* < 0.017; PIR–SDO, *F*_ST_= 0.655, *P* < 0.017).

Bottleneck analysis showed an excess of *H*_o_ in relation to *H*_eq_, which is a signal of population expansion after a drastic size reduction for the three mutation model (*P*≤ 0.001). Coalescent analysis also showed population expansion but not for population SDO (Table 3). Mutation parameter (θ) was low in all populations and migration among populations was negligible (Table 2). Using the coalescent parameter overall population (θ= 1.505), we were able to estimate the TMRCA, which dated to 50,166.67 year BP (95% CI = 28,666.67–86,000.00 year BP).

## Discussion

Our results showed that the disjunction distribution of *T. papyrus* is most likely due to the range contraction of an ancient more widely distribution, representing a climatic relict of drier and colder ice ages in Central and Southeast Brazil.

Populations of *T. papyrus* are highly differentiated for both, chloroplast and microsatellite markers. High differentiation is usually found in endemic species (e.g., Collevatti et al. 2009a; Zhou et al. 2010; Ge et al. 2011; Jones and Gibson 2011; Wang et al. 2011). Pollination and dispersal systems of *T. papyrus* may also foster population isolation and differentiation. This species is pollinated by large bees, and seeds are very small and dispersed by autochory. Although large bees may fly long distances (Goulson and Stout 2001), the patchy distribution of *T. papyrus* may favor the isolation of pollinators on patches of plants, restricting pollen flow due to long distance among suitable habitats. Also, the small seeds of *T. papyrus* are not winged and might not fly long distances, which may also pose limitations on the expansion of populations by diffuse or jump dispersal. Thus, pollen dispersal most likely contributes more to long distance dispersal than seeds. Dispersal may be constrained because the species is unable to cross barriers or because species is habitat specialist and may not succeed in habitats available in the barrier (Cox and Moore 2005). *Tibouchina papyrus* life history suggests that both factors may constrain species expansion. Hence, a modification in habitat availability should be necessary for species geographical distribution expansion.

Despite the low mutation parameter θ, populations presented high haplotype diversity for chloroplast genome. This is most likely due to the historical constant population size with no signal of severe reduction in population size, which maintained diversity despite low mutation rate. However, analyses of microsatellite loci showed a different demographical history and lower diversity than that usually found for microsatellite loci in savanna trees (e.g., Collevattti et al. 2001, Braga et al. 2007; Pereira et al. 2008; Rabelo et al. 2011). In fact, analyses on demographic history for microsatellite loci showed a reduction in population size that was not detected by chloroplast genome. This is most likely due to the differences in mutation rate and the time since the bottleneck event occurred (see Excoffier et al. 2009 for a review). Because microsatellite loci display high mutation rates, typically in the range of 10^–3^ to 10^–4^ per locus per generation (see Goldstein and Schlotterer 1999 for a review), they are more prone to the effects of recent demographic events. Those differences are denoted in the TMRCA, which was lower for microsatellite loci than for chloroplast genome. Besides differences in mutation rate, this may also be the outcome of a more recent bottleneck that may not be detected by a region with slower evolutionary rate. As coalescence of microsatellites which evolves faster coincides with ∼50 kyr BP (∼28–86 kyr BP), that predates the last glacial maximum (LGM), we hypothesize that populations suffered a bottleneck during the Sangamonian interglacial period (∼110–130 kyr BP) and started to expand during the last glaciation (∼12–110 kyr BP). Also, because the power to detect expansion after a reduction in population size is dependent on time since population reduction when using neutrality test or mismatch distribution (Excoffier 2004; Excoffier et al. 2009), ancient demographic expansion may be undetectable in both chloroplast genome and microsatellite loci. However, major coalescence times (see Fig. 3) coincided with pre-Illinoian period, specially the Cromerian interglacial (∼455–620 kyr BP) which may indicate periods of smaller effective population size.

Network analysis indicated that the haplotype H7 (Fig. 2) from Serra dos Pirineus population is the extant more ancient haplotype and despite the distance between Serra dos Pirineus and Serra Dourada, the ancestor of the last population diverged first (Fig. 3). This result may be the outcome of limited genome sampling leading to errors in coalescence time estimation and should be interpreted with conscious. However, despite the difference between the median-joining network based on parsimony criterion and on Bayesian coalescent tree, the clade comprising the lineages of PIR population has the lower number of mutations and thus the lowest number of character changing, suggesting that PIR is the ancestral population of *T. papyrus.* Hence, we hypothesize a bidirectional population expansion from Serra dos Pirineus toward the Southeast and Northwest during the glacial periods. This region of Central Brazil is comprised by many highlands, such as Chapada Pratinha, Chapada dos Veadeiros that could have connected PIR population to Serra da Natividade, the Northern distribution of *T. papyrus*.

Although estimations presented high variation, the TMRCA for populations dates from the Pre-Illinoian Cromerian interglacial that occurred between 620,000 and 455,000 year BP. This interglacial was followed by the Anglian Glaciation (Pre-Illinoian), that last from 455,000 to 300,000/380,000 year BP (Gibbard and Kolfschoten 2004; Gibbard et al. 2007). In the last glacial period, the Wisconsin Glaciation (∼110,000 to 12,000 year BP), the expansion and changing in geographical distribution is observed in the fossil records for some species of high altitudes, such as *Podocarpus* (e.g., Colinvaux et al. 1996), *Aracauria*, and other conifer taxa (Ledru 1993). Based on the above scenario, we hypothesize that *T. papyrus* expanded its distribution in the glacial periods of the Pre-Illinoian or Cromerian complex, favored by the colder and drier conditions that created suitable habitats. With the warmer and moisture conditions of the following interglacials, many populations extinguished and *T. papyrus* became restricted to drier quartizite and sandstone soils, attaining the present distribution in the Central Brazil. Thus, the present disjunct distribution may represent a climatic relict of an ancient widely distributed population. Indeed, the high differentiation and low gene flow between populations are also evidences that vicariance is responsible for the disjunct distribution. This same process was also suggested for *Lychnophora ericoides* (Asteraceae), a species from rocky savannas that co-occurs with *T. papyrus* in SDO and PIR (Collevatti et al. 2009a), despite the more recent TMRCA of *L. ericoides*. The differences in TMRCA are most likely due to the more wide geographical distribution of *L. ericoides* in the rocky savannas of the Central Brazil and differences in demographic parameters and ongoing gene flow among populations (Collevatti et al. 2009a).

In conclusion, our results strongly support that the disjunct distribution of *T. papyrus* may represent a climatic relict and that long distance gene flow is unlikely. *Tibouchina papyrus* has undergone a recent bottleneck that may have caused loss in genetic diversity. Since migration among populations is negligible, the probability of local extinction due to demographic and genetic stocasticity may be very high. *Tibouchina papyrus* is a rare and endemic species of sandstone outcrop habitat, which is highly unstable and suffers high levels of disturbance caused by fire during the dry season and sandstone and quartzite disruption, mainly during the rainy season. These disturbances may be highly variable among local habitats and may cause sudden modifications in population size (Collevatti et al. 2010). An important outcome of our study is the evidence that *T. papyrus* populations are three different ESUs (Evolutionary Significant Units, Moritz 1994) that may have adaptive differences. Hence, these populations require separate genetic management. These results are highly important for planning and executing scientifically sound conservation programs for the species. Moreover, due to remarkable differentiation and ancient divergence of the three lineages from the three localities, we suggest a more in depth taxonomical study of this species to review its classification in only one species. Finally, the present work showed remarkable concordance in the mechanisms involved in the evolution of the disjunct distribution of two unrelated species from rocky savanna, despite differences in some demographic parameters. This result is a strong evidence for the hypothesis raised in this work for the evolution of disjunct distribution in plant species from rocky savannas in Central Brazil.

## Supporting Information - Evolutionary Analysis

**Table S1.** Haplotypes of *Tibouchina papyrus* basedon the combined chloroplast DNA data from *psbA-trnH*,*trnC-ycf6*, and on *trnS-trnG*. *N*-total number ofindividuals for each haplotype.

## References

[b1] Arbogast BS, Edwards SV, Wakeley J, Beerli P, Slowinski JB (2002). Estimating divergence times from molecular data on phylogenetic and population genetic timescale. Annu. Rev. Ecol. Syst.

[b2] Azuma H, García-Franco JG, Rico-Gray V, Thien LB (2001). Molecular phylogeny of the Magnoliaceae: the biogeography of tropical and temperate disjunctions. Am. J. Bot.

[b3] Bandelt HJ, Forster P, Röhl A (1999). Median-joining networks for inferring intraspecific phylogenies. Mol. Biol. Evol.

[b4] Behling H (1995). Investigations into the Late Pleistocene and Holocene history of vegetation and climate in Santa Catarina (S Brazil). Veg. His. Archaeobot.

[b5] Behling H (2003). Late glacial and Holocene vegetation, climate and fire history inferred from Lagoa Nova in the southeastern Brazilian lowland. Veg. Hist. Archaeobot.

[b6] Behling H, Hooghiemstra H, Markgraf V (2000). Neotropical savanna environments in space and time: late quaternary. Interhemispheric climate linkages.

[b7] Braga AC, Reis AMM, Leoi LT, Pereira RW, Collevatti RG (2007). Development and characterization of microsatellite markers for the tropical tree species *Tabebuia aurea* (Bignoniaceae). Mol. Ecol. Notes.

[b8] Clegg MT, Gaut BS, Learn JRGH, Morton BR (1994). Rates and patterns of chloroplast DNA evolution. Proc. Natl. Acad. Sci.

[b9] Cockerham CC (1969). Variance of gene frequencies. Evolution.

[b10] Colinvaux PA, Oliveira P, Moreno JE, Miller MC, Bush MB (1996). A long pollen Record from Lowland Amazonia: Forest and cooling in glacial times. Science.

[b11] Collevatti RG, Grattapaglia D, Hay JD (2001). Population genetic structure of the endangered tropical tree species *Caryocar brasiliense*based on variability at microsatellite loci. Mol. Ecol.

[b12] Collevatti RG, Grattapaglia D, Hay JD (2003). Evidences for multiple maternal lineages of *Caryocar brasiliense* populations in the Brazilian Cerrado based on the analysis of chloroplast DNA sequences and microsatellite haplotype variation. Mol. Ecol.

[b13] Collevatti RG, Leoi LCT, Leite SA, Gribel R (2009b). Contrasting patterns of genetic structure in *Caryocar* (Caryocaraceae) congeners from flooded and upland Amazonian forests. Biol. J. Linn. Soc.

[b14] Collevatti RG, Lima JS, Soares TN, Telles MPC (2010). Spatial genetic structure and life history traits in cerrado tree species: inferences for conservation. Nat. Conserv.

[b15] Collevatti RG, Rabelo SG, Vieira RF (2009a). Phylogeography and disjunct distribution in *Lychnophora ericoides* (Asteraceae), an endangered cerrado shrub species. Ann. Bot.

[b16] Cornuet JM, Luikart G (1997). Description and power analysis of two tests for detecting recent population bottlenecks from allele frequency data. Genetics.

[b17] Cox CB, Moore PD (2005). Biogeography: an ecological and evolutionary approach.

[b18] Creste S, Tulmann-Neto A, Figueira A (2001). Detection of Single sequence repeat polymorphisms in denaturing polyacrylamide sequencing gels by silver staining. Plant Mol. Biol. Rep.

[b19] Davis MB, Shaw RG (2001). Range shifts and adaptive responses to Quaternary climate change. Science.

[b20] Demesure B, Comps B, Petit R (1996). Chloroplast DNA phylogeography of the common beech (*Fagus sylvatica* L.) in Europe. Evolution.

[b21] Dick CW, Abdul-Salim K, Bermingham E (2003). Molecular systematics reveals cryptic tertiary diversification of a widespread tropical rainforest tree. Am. Nat.

[b22] Dick CW, Bermingham E, Lemes MR, Gribel R (2007). Extreme long-distance dispersal of the lowland tropical rainforest tree *Ceiba pentandra* L. (Malvaceae) in Africa and the Neotropics. Mol. Ecol.

[b23] Doyle JJ, Doyle JL (1987). Isolation of plant DNA from fresh tissue. Focus.

[b24] Drummond AJ, Rambaut A (2007). BEAST: bayesian evolutionary analysis by sampling trees. BMC Evol. Biol.

[b25] Ellegren H (2000). Microsatellite mutations in the germline: implications for evolutionary inference. Trends Genet.

[b26] Ennos RA (1994). Estimating the relative rates of pollen and seed migration among plant populations. Heredity.

[b27] Excoffier L (2004). Patterns of DNA sequence diversity and genetic structure after a range expansion: lessons from the infinite-island model. Mol. Ecol.

[b28] Excoffier L, Foll M, Petit RJ (2009). Genetic consequences of range expansions. Annu. Rev. Ecol. Evol. Syst.

[b29] Excoffier L, Laval G, Schneider S (2005). Arlequin ver. 3.0: an integrated software package for population genetics data analysis. Evol. Bioinf. Online.

[b30] Excoffier L, Smouse P, Quattro J (1992). Analysis of molecular variance inferred from metric distances among DNA haplotypes: Application to human mitocondrial DNA restriction data. Genetics.

[b31] Forster P, Bandelt HJ, Röhl A (2004). http://www.fluxus-engineering.com.

[b32] Furley PA, Ratter JA (1988). Soil resource and plant communities of the central Brazilian Cerrado and their development. J. Biogeogr.

[b33] Gaudeul M (2006). Disjunct distribution of *Hypericum nummularium* L. (Hypericaceae): molecular data suggest bidirectional colonization from a single refugium rather than survival in distinct refugia. Biol. J. Linn. Soc.

[b34] Ge XJ, Hwang CC, Liu ZH, Huang CC, Huang WH, Hung KH, Wang WK, Chiang TY (2011). Conservation genetics and phylogeography of endangered and endemic shrub *Tetraena mongolica* (Zygophyllaceae) in Inner Mongolia, China. BMC Genet.

[b35] Gibbard P, Kolfschoten TV, Gradstein FM, Ogg JG, Smith AG (2004). The pleistocene and holocene epochs. A geologic time scale 2004.

[b36] Gibbard PL, Boreham S, Cohen KM, Moscariello A (2007). Global chronostratigraphical correlation table for the last 2.7 million years v. 2007b.

[b37] Goldstein DB, Schlotterer C (1999). Microsatellites: evolution and applications.

[b38] Goodman SJ (1997). RST Calc: a collection of computer programs for calculating estimates of genetic differentiation from microsatellite data and determining their significance. Mol. Ecol.

[b39] Goudet J (2002). FSTAT 2.9.3.2, a program to estimate and test gene diversities and fixation indices. http://www.unil.ch/izea/softwares/fstat.html.

[b40] Goudet J, Raymond M (1996). Testing differentiation in diploid population. Genetics.

[b41] Goulson D, Stout JC (2001). Homing ability of the bumblebee *Bombus terrestris* (Hymenoptera: Apidae). Apidologie.

[b42] Hardy OJ, Charbonnel N, Fréville H, Heuertz M (2003). Microsatellite allele sizes: a simple test to assess their significance on genetic differentiation. Genetics.

[b43] Hardy OJ, Vekemans X (2002). Spagedi: a versatile computer program to analyze spatial genetic structure at the individual or population levels. Mol. Ecol. Notes.

[b44] Hewitt GM (1996). Some genetic consequences of ice ages, and their role in divergence and speciation. Biol. J. Linn. Soc.

[b45] Hurlbert SH (1971). The nonconcept of species diversity: a critique and alternative parameters. Ecology.

[b46] Jones JM, Gibson JP (2011). Population genetic diversity and structure within and among disjunct populations of *Alnus maritima* (seaside alder) using microsatellites. Conserv. Genet.

[b47] Karanth KP (2003). Evolution of disjunct distributions among wet-zone species of the Indian subcontinent: testing various hypotheses using a phylogenetic approach. Curr. Sci.

[b48] Kingman JFC (1982a). The coalescent. Stochastic Process. Appl.

[b49] Kingman JFC (1982b). On the genealogy of large populations. J. Appl. Probab.

[b50] Kuhner MK (2006). LAMARC 2.0: maximum likelihood and Bayesian estimation of population parameters. Bioinformatics.

[b51] Kuhner MK, Smith LP (2007). Comparing likelihood and Bayesian coalescent estimation of population parameters. Genetics.

[b52] Ledru MP (1993). Late Quaternary environmental and climatic change in central Brazil. Quat. Res.

[b53] Lorenz-Lemke AP, Togni PD, Mäder G, Kriedt RA, Stehmann JR, Salzano FM, Bonatto SL, Freitas LB (2010). Diversification of plant species in a subtropical region of eastern South American highlands: a phylogeographic perspective on native *Petunia* (Solanaceae). Mol. Ecol.

[b54] Luikart GL, Allendorf FW, Cornuet JM, Sherwin WB (1998). Distortion of allele frequency distributions provides a test for recent population bottlenecks. J. Hered.

[b55] Marriage TN, Hudman S, Mort ME, Orive ME, Shaw RG, Kelly JK (2009). Direct estimation of the mutation rate at dinucleotide microsatellite loci in *Arabidopsis thaliana* (Brassicaceae). Heredity.

[b56] Maruyama T, Fuerst PA (1985). Population bottlenecks and non-equilibrium models in population genetics. II. Number of alleles in a small population that was formed by a recent bottleneck. Genetics.

[b57] McCauley DE (1995). The use of chloroplast DNA polymorphism in studies of gene flow in plants. Trends Ecol. Evol.

[b58] Molero J, Daviña JR, Honfi AI, Franco D, Rovira A (2006). Chromosome studies on plants from Paraguay II. Candollea.

[b59] Montoro GR, Santos ML (2007). Fenologia e Biologia Reprodutiva de *Tibouchina papyrus* (Pohl.) Toledo no Parque Estadula da Serra dos Pireneus, Goiás. Rev. Biol. Neotrop.

[b60] Moritz C (1994). Defining “Evolutionarily Significant Units” for conservation. Trends Ecol. Evol.

[b61] Mousakik AE, Petit RJ (1996). Chloroplast DNA phylogeography of the argan tree of Morocco. Mol. Ecol.

[b62] Nei M (1978). Estimation of average heterozygosity and genetic distance from a small number of individual. Genetics.

[b63] Nei M (1987). Molecular evolutionary genetics.

[b64] Pereira MF, Bandeira LF, Blanco AJV, Ciampi AY, Coelho ASG (2008). Development of microsatellite markers in *Annona crassiflora* Mart., a Brazilian Cerrado fruit tree species. Mol. Ecol. Resour.

[b65] Petit RJ, Mousakik AE, Pons O (1998). Identifying populations for conservation on the basis of genetic markers. Conserv. Biol.

[b66] Rabelo SG, Teixeira CF, Telles MPC, Collevatti RG (2011). Development and characterization of microsatellite markers for *Lychnophora ericoides*an endangered Cerrado shrub species. Conserv. Genet. Resour.

[b67] Rambaut A, Drummond AJ (2007). http://beast.bio.ed.ac.uk/Tracer.

[b68] Ramos ACS, Lemos-Filho JP, Ribeiro RA, Santos FR, Lovato MB (2007). Phylogeography of the tree *Hymenaea stigonocarpa* (Fabaceae: Caesalpinioideae) and the influence of Quaternary climate changes in the Brazilian Cerrado. Ann. Bot.

[b69] Ribeiro JF, Walter BMT, Sano SM, Almeida SP (1998). Fitofisionomias do Bioma Cerrado. Cerrado: ambiente e flora.

[b70] Roger A, Harpending HC (1992). Population growth makes waves in the distribution of pairwise genetic differences. Mol. Biol. Evol.

[b71] Salgado-Labouriau ML, Barberi M, Ferraz-Vicentini KR, Parizzi MG (1998). A dry climatic event during the late Quaternary of tropical Brazil. Rev. Paleobot. Palynol.

[b72] Schaal BA, Hayworth DA, Olsen KM, Rausher JT, Smith WA (1998). Phylogeographic studies in plants: problems and prospects. Mol. Ecol.

[b73] Schneider S, Excoffier L (1999). Estimation of demographic parameters from the distribution of pairwise differences when the mutation rates vary among sites: application to human mitochondrial DNA. Genetics.

[b74] Shaw J, Lickey EB, Beck JT, Farmer SB, Liu W, Miller J, Siripun KC, Winder CT, Schilling EE, Small RL (2005). The tortoise and the hare II: relative utility of 21 noncoding chloroplast DNA sequences for phylogenetic analysis. Am. J. Bot.

[b75] Slatkin M (1995). A measure of population subdivision based on microsatellite allele frequencies. Genetics.

[b76] Smith SA, Donoghue MJ (2008). Rates of molecular evolution are linked to life history in flowering plants. Science.

[b77] Stehlik I, Holderegger R, Schneller JJ, Abbott RJ, Bachmann K (2000). Molecular biogeography and population genetics of alpine plant species. Bull. Geobot. Inst. ETH.

[b78] Tajima F (1989). Statistical method for testing neutral mutation hypothesis by DNA polymorphism. Genetics.

[b79] Teixeira AH (1969). Árvore do papel *Tibouchina papyrus* (Pohl) Toledo. Anais XX Congr. Nac. Soc. Bot. Brasil.

[b80] Telles MPC, Peixoto FP, Lima JS, Resende LV, Vianello RP, Walter MEMT, Collevatti RG (2011). Development of microsatellite markers for the endangered Neotropical tree species *Tibouchina papyrus* (Melastomataceae). Genet. Mol. Res.

[b81] Thompson JD, Gibson TJ, Plewniak F, Jeanmougin F, Higgins DG (1997). The ClustalX windows interface: flexible strategies for multiple sequence alignment aided by quality analysis tools. Nucleic Acids Res.

[b82] Thuillet AC, Bru D, David J, Roumet P, Santoni S, Sourdille P, Bataillon T (2002). Direct estimation of mutation rate for 10 microsatellite loci in durum wheat, *Triticum turgidum* (L.) Thell. ssp *durum* Desf. Mol. Biol. Evol.

[b83] Udupa SM, Baum M (2001). High mutation rate and mutational bias at (TAA)n microsatellite loci in chickpea (*Cicer arietinum* L.). Mol. Genet. Genomics.

[b84] Vigouroux Y, Jaquet JSH, Matsuoka Y, Smith OS, Beavis WD, Smith JS, Doebley J J (2002). Rate and pattern of mutation at microsatellite loci in maize. Mol. Biol. Evol.

[b85] Wang JF, Pan YZ, Gong X, Chiang YC, Kuroda C (2011). Chloroplast DNA variation and phylogeography of *Ligularia tongolensis* (Asteraceae), a species endemic to the Hengduan Mountains Region of China. J. Syst. Evol.

[b86] Weir BS, Cockerham CC (1984). Estimating F-statistics for the analysis of population structure. Evolution.

[b87] Wolfe KH, Li WH, Sharp PM (1987). Rates of nucleotide substitution vary greatly among plant mitochondrial, chloroplast, and nuclear DNAs. Proc. Natl. Acad. Sci.

[b88] Wright S (1951). The genetical structure of populations. Ann. Eugen.

[b89] Yamane K, Yano K, Kawahara T (2006). Pattern and rate of indel evolution inferred from whole chloroplast intergenic regions in sugarcane, maize and rice. DNA Res.

[b90] Zhou TH, Li S, Qian ZQ, Su HL, Huang ZH, Gou ZG, Dai PF, Liu ZL, Zhao GF (2010). Strong phylogeographic pattern of cpDNA variation reveals multiple glacial refugia for *Saruma henryi* Oliv. (Aristolochiaceae), an endangered herb endemic to China. Mol. Phylogenet. Evol.

