# Supplementary material for: Phylogeography of Tibouchina papyrus (Pohl) Toledo (Melastomataceae), an endangered tree species from rocky savannas, suggests bidirectional expansion due to climate cooling in the Pleistocene
Source: Ecol Evol. 2012 May;2(5):1024–35. doi: 10.1002/ece3.236 (PMC3399167; doi:10.1002/ece3.236)
Supplement: Supplementary file 1 [file ece30002-1024-SD1.doc]

**Appendix – Supplemental Material**

S1. Haplotypes of *Tibouchina papyrus* based on the combined chloroplast DNA data from *psbA-trnH*, *trnC-ycf6* and on *trnS-trnG*. N – total number of individuals for each haplotype.

|  | **Combined cpDNA** | | | | | | | | | | | | | | |  | | | |
| --- | --- | --- | --- | --- | --- | --- | --- | --- | --- | --- | --- | --- | --- | --- | --- | --- | --- | --- | --- |
|  | *psbA/trnH* | | | | |  | | *trnC/ycf6* | | |  | | *trnS/trnG* | | | | | |  |
| **Haplotype** | 1  2  3 | 1  7  6 | 2  1  0 | 2  2  3 | 2  2  6 | |  | | 4  1  3 | 5  0  7 | |  | | 6  2  9 | 8  6  7 | | 8  8  6 | 1  0  3  1 | **N** |
| H01 | – | T | C | G | C | |  | | C | T | |  | | C | T | | A | G | 02 |
| H02 | – | . | . | . | . | |  | | . | G | |  | | . | . | | . | . | 17 |
| H03 | – | G | . | . | . | |  | | A | . | |  | | . | . | | . | . | 12 |
| H04 | – | G | . | . | . | |  | | . | G | |  | | . | . | | . | . | 01 |
| H05 | T | G | . | . | . | |  | | A | . | |  | | . | C | | – | . | 09 |
| H06 | T | G | . | T | . | |  | | A | . | |  | | . | C | | – | . | 10 |
| H07 | – | G | . | . | . | |  | | A | . | |  | | . | . | | – | . | 09 |
| H08 | – | G | . | T | . | |  | | A | . | |  | | . | . | | – | . | 01 |
| H09 | T | G | . | . | . | |  | | A | . | |  | | . | . | | – | . | 01 |
| H10 | – | G | . | . | . | |  | | A | . | |  | | . | C | | – | . | 02 |
| H11 | – | G | . | . | . | |  | | A | . | |  | | T | . | | – | . | 08 |
| H12 | – | G | . | . | A | |  | | A | . | |  | | T | . | | – | . | 08 |
| H13 | – | G | . | . | . | |  | | A | . | |  | | T | . | | – | T | 06 |
| H14 | – | G | G | . | A | |  | | A | . | |  | | T | . | | – | . | 03 |
| H15 | – | G | G | . | . | |  | | A | . | |  | | T | . | | – | . | 02 |
| H16 | T | G | . | . | . | |  | | A | . | |  | | T | . | | – | . | 01 |
| H17 | – | G | . | . | A | |  | | A | . | |  | | T | . | | – | T | 04 |
